# An Australian Parliament and a Charities Bill

**Published:** 1913-02-08

**Authors:** 


### AN AUSTRALIAN PARLIAMENT AND A CHARITIES BILL.

### The Managers' Attitude to its Proposals.

BY OUR AUSTRALIAN CORRESPONDENT.

Amongst the "slaughtered innocents" for the second consecutive session of the Victorian State Parliament is included "A Bill to Consolidate and Amend the Law relating to Hospitals and Charities."

This Bill reached a second-reading stage, but though put forward by the Government as a non-party measure, the prospects of its emerging from Committee, save in a mutilated form, were so remote that the State Premier, the Hon. W. A. Watt, M.L.A., reluctantly abandoned it in the last week of the session. The gist of the Bill was the creation of a Board of three well-paid Commissioners, with the inevitable retinue of officials, the former to be elected by the Governor in Council, and who, as a Board, were, subject to the provisions of the Act, to determine: (*a*) That any subsidised institution not being a "separate institution" shall be closed; (*b*) that any two or more subsidised institutions not being "separate institutions" shall be amalgamated; (*c*) the purposes for which any subsidised institution not being a "separate institution" shall be used; (*d*) that any subsidised benevolent society shall cease to exist; (*e*) give from time to time to any subsidised benevolent societies such directions as it thinks fit with a view to systematising the work; (*f*) attach any prescribed conditions to the payment to subsidised institutions or benevolent societies of any sums out of the Fund; or (*g*) withhold payment to any subsidised institution or benevolent society. Also, in addition to powers conferred by the Act, "the Board shall have and exercise all such other powers and duties as are conferred or imposed upon it from time to time by the Governor in Council."

It should be explained to English readers that "separate institutions" included all those under the management and control of religious bodies to the number of ten, one being the St. Vincent's Roman Catholic Hospital, containing over 130 beds. Further, this Board of three were to be empowered to establish and wholly maintain intermediate hospitals for persons who were not rich enough to pay for treatment in private hospitals and not poor enough to be treated in a public charity. And again, to quote from this printed Bill, which runs to thirty pages and includes eighty distinct clauses. "Notwithstanding anything contained in this or any other Act or in any by-laws or regulations, no subsidised institution shall erect any building or make any structural alteration of or addition to any building or premises involving an expenditure exceeding one hundred pounds without the previous approval in writing of the Board." This means that if a benevolent individual gave or bequeathed over £100 to a hospital for some specific purpose, involving alterations or additions to existing buildings, the Government Board might veto this expenditure and there could be no appeal against the exercise of such arbitrary power.

Small wonder, therefore, in view of these and other equally drastic and objectionable clauses, that the committee of nearly every large hospital in the State were up in arms, and argued that if such a measure became law no self-respecting man or woman would remain on the management of charitable institutions.

